# Lower lipoprotein(a) levels in patients with acute lacunar infarction

**DOI:** 10.1016/j.ibneur.2026.09.001

**Published:** 2026-09-08

**Authors:** Xiangliang Chen, Nihong Chen, Lin Zhu, Qing Huang, Fuping Jiang

**Affiliations:** a Department of Neurology, Nanjing First Hospital, Nanjing Medical University, Nanjing, 210006, China; b Department of Geriatrics, Nanjing First Hospital, Nanjing Medical University, Nanjing, 210006, China

**Keywords:** Lipoprotein(a), Acute lacunar infarction, Cerebral small vessel disease

## Abstract

**Background:**

Serum lipoprotein(a) [Lp(a)] is a well-established risk factor for atherosclerotic cardiovascular diseases. However, studies on the correlation between Lp(a) and acute lacunar infarction (ALI) remain inconclusive. We conducted a case-control study to investigate the association between serum Lp(a) levels and ALI in a Chinese population.

**Methods:**

We consecutively enrolled 989 patients with ALI (719 males, 270 females; mean age 64.63 ± 11.97 years) and an equal number of sex-matched controls with available brain MRI from the Departments of Neurology and Geriatrics. Univariate and multivariable binary logistic regression analyses were used to examine the relationships between Lp(a) concentrations and the presence of ALI.

**Results:**

Serum Lp(a) levels were significantly lower in the ALI group compared to controls. Multivariable logistic regression revealed an independent inverse association between Lp(a) levels and the presence of ALI (odds ratio [OR], 0.76; 95% confidence interval [CI], 0.66–0.87; *P* < 0.001. The trend held across age and sex subgroups (age ≥ 60 years: OR, 0.71; 95% CI, 0.60–0.84; *P* < 0.001; age < 60 years: OR, 0.76; 95% CI, 0.59–0.98; *P* = 0.034; males: OR, 0.80; 95% CI*,* 0.69–0.94; *P* = 0.007; females: OR, 0.68; 95% CI, 0.53–0.88; *P* = 0.003). For lesion locations, inverse associations existed in the basal ganglia and brainstem/cerebellum lesions, but not in the subcortical white matter.

**Conclusions:**

This study demonstrates that lower serum Lp(a) levels are independently associated with the presence of ALI. The clinical implications and underlying biological mechanisms of this observation warrant further investigation.

## Highlights

• Lower Lp(a) independently associates with acute lacunar infarction.
• This inverse link remains consistent across age and sex subgroups.
• Negative link is found in the basal ganglia and brainstem/cerebellum.
• No Lp(a) association was seen for the infarcts located in the subcortical white matter.

## 1 Introduction

Acute lacunar infarction (ALI) refers to a small noncortical infarction resulting from the occlusion of a single penetrating artery in the deep cerebral hemisphere or brainstem(Arauz et al., 2003; Wardlaw, 2005). ALI belongs to the spectrum of cerebral small vessel disease (CSVD)(Caplan, 2015), and its pathogenesis is heterogeneous, including hyaline degeneration, fibrinoid necrosis, amyloidosis, micro-embolism, and micro-atherosclerosis(Markus and de Leeuw, 2023; Regenhardt et al., 2018). In contrast, cerebral infarction due to large artery atherosclerosis primarily follows the disease process of atheromatous plaque formation, stenosis, or occlusion of a major extracranial or intracranial artery, driven by a single pathogenic mechanism: atherosclerosis. Given such fundamental differences in pathogenesis, emerging evidence indicates that the risk factors for the two are not completely identical(Feng et al., 2013; Giroud et al., 1992; Jackson and Sudlow, 2005; Jackson et al., 2010; Schilling et al., 2014; Shi et al., 2021; Shu et al., 2021): hypertension, diabetes, hypertriglyceridemia, and smoking are common risk factors for both(Jackson and Sudlow, 2005; Jackson et al., 2010; Shi et al., 2021; Shu et al., 2021), but hypercholesterolemia is more likely to cause lacunar infarction(Jackson and Sudlow, 2005; Shi et al., 2021), while atrial fibrillation has a stronger association with large artery atherosclerosis(Jackson and Sudlow, 2005; Jackson et al., 2010).

Lipoprotein(a) [Lp(a)] is a lipid particle similar to low-density lipoprotein (LDL). It consists of an LDL-like molecule and a variable-length apolipoprotein (a) [Apo(a)], which is covalently bound to ApoB100 through a single disulfide bridge(Koschinsky et al., 1993). The concentration of Lp(a) in the population is mainly influenced by genetic factors(Milionis et al., 2000), with more than 90% of interindividual variation determined by polymorphisms at the *LPA* locus(Kronenberg, 2014). Circulating Lp(a) levels remain highly stable throughout a person's life and are largely unaffected by age, body weight, diet, exercise, and traditional lipid-lowering drugs (Hobbs and White, 1999, Kronenberg et al., 2022). Notably, Lp(a) concentrations also appear resistant to acute fluctuations triggered by inflammation, stress or acute ischemic stroke(Byrne et al., 2002; Kargman et al., 1998).

The pathophysiological role of Lp(a) remains incompletely understood. A large number of studies have shown that Lp(a) accelerates atherosclerotic progression, establishing it as a risk factor for atherosclerotic cardiovascular diseases(Bhatia et al., 2025; Clarke et al., 2009) as well as for carotid atherosclerosis(Duan et al., 2023; van Dam-Nolen et al., 2021). However, evidence of the relationship between Lp(a) and overall ischemic stroke is equivocal, with some studies linking elevated Lp(a) levels to an increased risk of ischemic stroke, whereas others have detected no significant association(Glader et al., 1999; Langsted et al., 2019; Larsson et al., 2020; Nave et al., 2015; Price et al., 2001; Ridker et al., 1995; Smolders et al., 2007). Currently, mounting evidence indicates that Lp(a) is a risk factor for large artery atherosclerosis-related cerebral infarction(Cerrato et al., 2002; Kumar et al., 2021), while studies exploring the correlation between Lp(a) and ALI are few and inconsistent(Cerrato et al., 2002; Petersen et al., 2007; Tang and Geng, 2019).

Intriguingly, a recent large-scale two-sample Mendelian randomization study by Pan et al. revealed a strong causal relationship between Lp(a) levels and various neurological conditions. The study found that the genetically predicted 1-SD increase in log-transformed Lp(a) concentration in European individuals was associated with a 20% increased risk of large artery stroke, but with an 8% reduced risk of small vessel stroke and a 6% reduced risk of Alzheimer disease. These findings were broadly consistent across multiple sensitivity analyses(Pan et al., 2019). However, this study was restricted to the database of Europeans, and the association between Lp(a) and ALI in Chinese individuals is yet to be elucidated. Accordingly, the present case-control study was performed to investigate the cross-sectional association serum Lp(a) levels and presence of ALI in a Chinese population.

## 2 Methods

### 2.1 Study design and participants

Consecutive patients admitted to the Department of Neurology or Geriatrics at Nanjing First Hospital of Nanjing Medical University between January 2016 and March 2021 were screened for the present study. The inclusion criteria were as follows: 1) age ≥ 18 years, 2) diagnosis of first-ever ALI confirmed by brain magnetic resonance imaging (MRI) with DWI hyper-intensity and corresponding ADC hypo-intensity. The exclusion criteria were as follows: 1) previous history of ischemic or hemorrhagic stroke; 2) incomplete clinical data; 3) brain malignancy.

During the same period, the same number of sex-matched adult patients without cerebral infarction confirmed by brain MRI were recruited from the same departments as the controls.

This study was conducted in accordance with the Declaration of Helsinki and was approved by the Ethics Committee of Nanjing First Hospital of Nanjing Medical University (Ethics approval number: KY20250611-KS-04).

### 2.2 Baseline characteristics

Demographic information, medical history, and laboratory data were collected for all participants. Comorbidities including coronary heart disease, atrial fibrillation, diabetes mellitus, and hypertension were identified through a review of medical records. Current smoking was defined as continuous tobacco use for the past six months or more(Xu et al., 2013). Current drinking was defined by daily consumption of alcohol ≥ 40 g for men and ≥ 20 g for women for a duration of more than 5 years(Zeng et al., 2008). Atrial fibrillation was diagnosed based on standard electrocardiographic criteria on at least one electrocardiogram(European Heart Rhythm et al., 2010). Diabetes mellitus was defined as fasting glucose ≥ 7.0 mmol/L, 2–hour post-load glucose ≥ 11.1 mmol/L, or glycosylated hemoglobin A1c (HbA1c) ≥ 6.5%, or current use of antidiabetic medications(Wang et al., 2017). Hypertension was defined as systolic blood pressure ≥ 140 mmHg, or diastolic blood pressure ≥ 90 mmHg, or current use of antihypertensive treatment(Wang et al., 2018).

Routine laboratory measurements included uric acid (UA), serum creatinine (Scr), blood urea nitrogen (BUN), fasting glucose (FG), total cholesterol (TC), triglycerides (TG), high-density lipoprotein cholesterol (HDL-C), low density lipoprotein cholesterol (LDL-C), HbA1c and Lipoprotein-associated phospholipase A2 (Lp-PLA2). Serum Lp(a) levels were measured by an immunoturbidimetric assay on an Abbott C16000 analyzer (Abbott Laboratories, Chicago, USA) with a measuring range of 10–3200 mg/L. Lp(a) concentrations were further categorized into five groups by quintiles as appropriate for analyses.

### 2.3 MRI acquisition and assessment

All eligible patients underwent brain MRI using a 3.0-T scanner (Ingenia, Philips Medical Systems) with an 8-channel receiver array head coil. The imaging protocol included T1-weighted axial images (repetition time [TR]/echo time [TE], 8.1/3.7 ms; slice thickness, 6 mm; matrix, 240 × 240), T2-weighted images (TR/TE, 7000/120 ms; slice thickness, 6 mm; matrix, 230 × 230), fluid-attenuated inversion recovery images (TR/TE, 7000 / 120 ms; slice thickness, 6 mm; matrix, 356 × 151), and diffusion-weighted images (TR/TE, 2501/98 ms; slice thickness, 6 mm; matrix, 152 × 122; b values of 0 and 1000 s/mm^2^)(Chen et al., 2021).

All MRI examinations were acquired during routine clinical care in the Department of Radiology at Nanjing First Hospital. ALI was defined as small subcortical infarcts (3–15 mm in diameter) in the territory of deep penetrating arteries, characterized by circularly-shaped hypo-intensity on T1WI, hyper-intensity on T2WI, and hyper-intensity on the fluid-attenuated inversion recovery sequence(Arauz et al., 2003; Wardlaw, 2005). All MRI images were independently reviewed by two experienced neuroradiologists who were blinded to clinical data. Inter-rater agreement between the two neuroradiologists was assessed and showed almost perfect consistency (kappa statistic 0.80–1.00). Any discrepancies were resolved by consensus review to reach a final diagnosis.

### 2.4 Statistical analysis

Categorical variables were presented as frequencies (percentages), and continuous variables as mean ± standard deviation (SD), as appropriate. The normality distribution of data was tested using the Kolmogorov-Smirnov test. Both the serum TG and Lp(a) concentrations showed right skewed distributions, and logarithmic transformation was applied to approximate normality. Baseline characteristics were compared between patients with and without ALI using Student’s *t* test for continuous variables (with log-transformed values for TG and Lp(a)) and chi-square test for categorical variables.

Multivariable binary logistic regression was utilized to examine the relationship between Lp(a) concentrations and the presence of ALI. For each model, Odds ratio (OR) and 95% confidence interval (CI) were calculated. Collinearity among candidate variables was assessed prior to the regression analyses using Spearman's or Pearson's correlation analyses as appropriate. A correlation coefficient *r* ≥ 0.7 indicated strong collinearity. Consequently, TC and lipid-lowering therapy were excluded from subsequent modeling due to the strong intercorrelations with LDL-C (TC vs. LDL: *r* = 0.883; lipid-lowering therapy vs. LDL: *r* = -0.864). HbA1c was also excluded, given its substantial correlations with FG and history of diabetes (*r* = 0.711 and 0.733, respectively). For each outcome, two adjusted models were conducted: model 1 adjusted for age and sex; model 2 further adjusted for histories of coronary artery disease, atrial fibrillation, hypertension, diabetes mellitus, current smoking, current drinking, year of enrollment, UA, BUN, Scr, FG, TG, LDL-C, HDL-C, and Lp-PLA2. The lgLp(a) was standardized to Z-scores in all regression models to facilitate per-1-SD effect interpretation.

To explore the pattern and magnitude of the relationship between Lp(a) and ALI, binary or ordinal logistic regression with restricted cubic splines for Lp(a) was performed. The Lp(a) level of 40 mg/L (33rd percentile) was set as the reference.

All statistical analyses and data visualization were performed with SPSS version 23 (IBM, Armonk, NY). A 2-tailed *P* value of < 0.05 was considered significant for all statistical tests.

## 3 Results

### 3.1 Baseline characteristics

A total of 989 MRI-confirmed ALI patients and 989 sex-matched controls were included in the present study. The proportion of males in both groups was 72.7%。The average ages of the ALI group and the control group were 64.63 ± 11.97 years and 65.45 ± 13.37 years, respectively. The main characteristics of the study population were summarized in Table 1. Compared with controls, patients with ALI had significantly higher values of FG (*P* < 0.001), TC (*P* = 0.04), TG (*P* = 0.01), LDL-C (*P* = 0.005), HbA1c (*P* < 0.001), and Lp-PLA2 (*P* < 0.001), but significantly lower values of Lp(a) (*P* < 0.001). Moreover, the ALI group had reported higher proportions of current smokers (*P* < 0.001) and current alcohol users (*P* < 0.001) and lower proportion of lipid-lowering therapy (*P* < 0.001). No significant between-group differences were observed in age, serum UA, Scr, BUN, HDL-C, diabetes mellitus, previous coronary artery disease or hypertension.

**Table 1:** Baseline characteristics according to the presence of ALI.

	**All subjects (n = 1978)**	**ALI (n = 989)**	**Controls (n = 989)**	**P value**
Age (years)	65.04 ± 12.69	64.63 ± 11.97	65.45 ± 13.37	0.15
UA (μmol/l)	326.25 ± 98.73	314.86 ± 99.03	336.10 ± 97.45	0.77
Scr (umol/l)	77.02 ± 40.89	75.08 ± 40.51	78.92 ± 41.19	0.64
BUN (mg/dl)	5.70 ± 2.22	5.43 ± 1.81	5.97 ± 2.53	0.33
FG (mmol/l)	5.82 ± 2.00	6.00 ± 2.36	5.64 ± 1.55	< 0.001
TC (mmol/l)	4.37 ± 1.05	4.46 ± 1.02	4.27 ± 1.08	0.04
lgTG (mmol/l)*	0.16 ± 0.24	0.18 ± 0.24	0.15 ± 0.25	0.02
HDL-C (mmol/l)	1.08 ± 0.28	1.06 ± 0.26	1.11 ± 0.30	0.15
LDL-C (mmol/l)	2.59 ± 0.86	2.71 ± 0.83	2.47 ± 0.88	0.01
lgLp(a) (mg/l)*	2.13 ± 0.37	2.07 ± 0.37	2.19 ± 0.36	< 0.001
HbA1c	6.26 ± 1.43	6.50 ± 1.64	6.01 ± 1.11	< 0.001
Lp-PLA2	170.71 ± 107.10	201.03 ± 125.33	141.02 ± 74.51	< 0.001
Sex, males, n (%)	1438 (72.7)	719 (72.7)	719 (72.7)	1.00
Coronary heart disease, n (%)	108 (5.46)	53 (5.36)	55 (5.56)	0.84
AF, n (%)	119 (6.02)	9 (0.91)	110 (11.12)	< 0.001
DM, n (%)	533 (26.95)	282 (28.51)	251 (25.38)	0.12
Hypertension, n (%)	1264 (63.95)	643 (65.02)	622 (62.90)	0.36
Current smoking, n (%)	850 (42.97)	523(52.88)	327 (33.06)	< 0.001
Current drinking, n (%)	566 (28.60)	383(38.73)	183 (18.50)	< 0.001
Lipid-lowering therapy, n (%)	1056 (53.39)	484 (48.94)	572 (57.84)	< 0.001

AF, atrial fibrillation; BUN, blood urea nitrogen; DM, diabetes mellitus; FG, fasting glucose; HbA1c, glycosylated hemoglobin A1c; HDL-C, high-density lipoprotein cholesterol; LDL-C, low-density lipoprotein cholesterol; Lp(a), lipoprotein(a); Scr, serum creatinine; TC, total cholesterol; TG, triglycerides; UA, uric acid；Lp-PLA2, Lipoprotein-associated phospholipase A2. *Lp(a) and TG were log-transformed to satisfy the normal distribution.

### 3.2 Association between Lp(a) levels and ALI

**Fig. 1:**
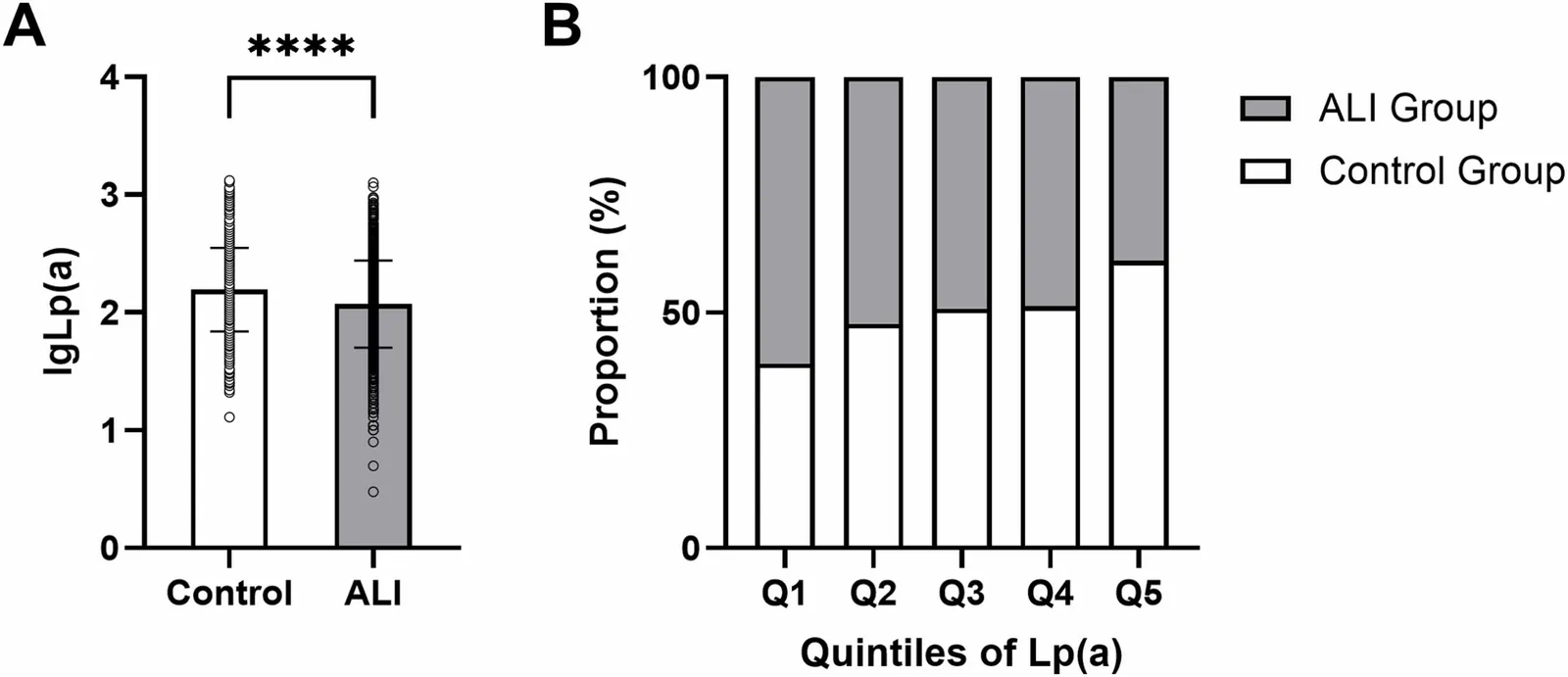
**Lp(a) levels and quintile distribution in ALI and control subjects.** (A) Bar chart showing log₁₀-transformed Lp(a) levels in the Control and ALI group. Data are presented as the means ± SD (n = 989); **** *P* < 0.0001 (Student’s *t* test). (B) Proportional distribution of Control and ALI subjects across Lp(a) quintiles.

Serum Lp(a) levels were significantly and negatively associated with ALI in both univariate and multivariate binary logistic regression analyses (all *P* < 0.001) **(**Fig. 1**;** Table 2**)**. The inverse association remained significant after adjustment for age and sex in Model 1 (*P* < 0.001) and persisted after further adjustment for previous coronary artery disease, atrial fibrillation, hypertension, diabetes mellitus, current smoking, current drinking, year of enrollment, UA, BUN, Scr, FG, TG, HDL-C, LDL-C, and Lp-PLA2 in Model 2 (*P* < 0.001). **(**Table 2**).**

**Table 2:** Binary logistic regression analysis for the association between Lp(a) level with ALI.

LgLp(a) ALI vs. control	Unajusted OR (95% CI)	*P* value	Model 1^a^		Model 2^b^		
			Ajusted OR (95% CI)	*P* value	Ajusted OR (95% CI)	*P* value	
2.07 ± 0.37 vs. 2.19 ± 0.36	0.71 (0.65–0.78)	< 0.001	0.71 (0.65–0.78)	< 0.001	0.76 (0.66–0.87)		< 0.001

a Model 1 adjusted for age and sex

b Model 2 adjusted for age, sex, coronary artery disease, atrial fibrillation, hypertension，diabetes mellitus, current smoking, current use of alcohol, year of enrollment, uric acid, serum creatinine, blood urea nitrogen, fasting glucose, triglycerides, low-density lipoprotein cholesterol, high density lipoprotein cholesterol, and Lipoprotein-associated phospholipase A2.

**Fig. 2:**
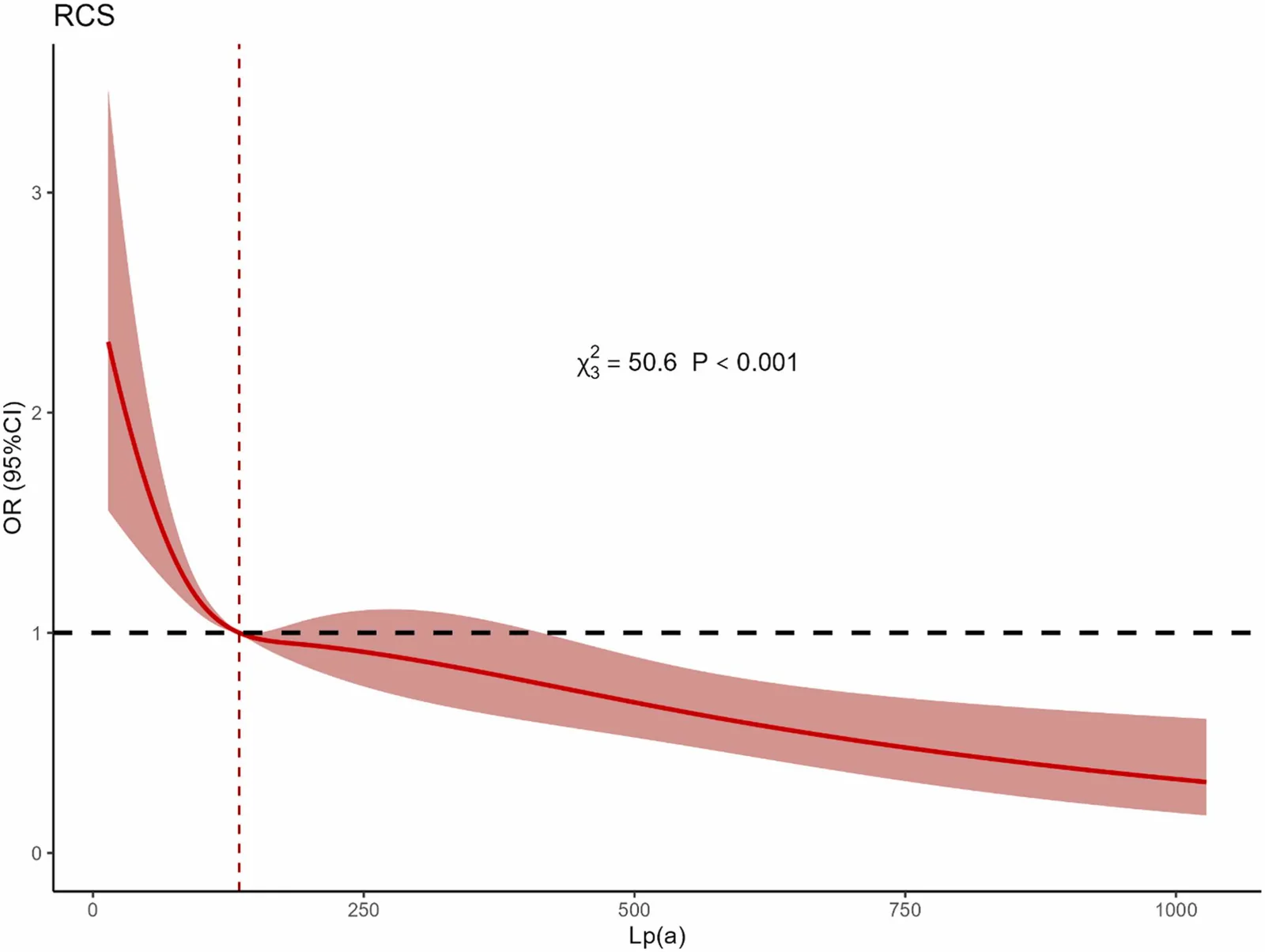
Restricted cubic spline analysis of the association between Lp(a) and ALI.

The binary logistic regression with restricted cubic spline curve demonstrated that higher serum Lp(a) levels had a lower probability of ALI, showing a consistent negative relationship with no evidence of an inflection point across the observed range. **(**Fig. 2**)**

### 3.3 Association between Lp(a) levels and ALI in different brain regions

The relationship between ALI and Lp(a) differed across brain regions **(**Table 3**)**. No significant correlation was detected for ALI located in the subcortical white matter. In contrast, significant inverse associations were observed for ALI in the basal ganglia region (Model 2: OR, 0.68; 95% CI, 0.58–0.80; *P* < 0.001) and the cerebellum and brainstem region (Model 2: OR, 0.76; 95% CI, 0.66–0.86, *P* < 0.001).

**Table 3:** The association between Lp(a) level with ALI in different brain regions.

Region of ALI	LgLp(a) ALI vs. control	Unadjusted OR (95% CI)	*P* value	Model 1^a^		Model 2^b^	
				Adjusted OR (95% CI)	*P* value	Adjusted OR (95% CI)	*P* value
Subcortical white matter	2.15 ± 0.40 vs. 2.20 ± 0.36	0.89 (0.75–1.05)	0.150	0.87 (0.73–1.03)	0.110	0.97 (0.77–1.25)	0.852
Basal ganglia	2.10 ± 0.39 vs. 2.20 ± 0.36	0.63 (0.56–0.71)	< 0.001	0.63 (0.57–0.71)	< 0.001	0.68 (0.58–0.80)	< 0.001
Cerebellum and brainstem	2.04 ± 0.35 vs. 2.20 ± 0.36	0.76 (0.66–0.88)	< 0.001	0.76 (0.65–0.88)	< 0.001	0.76 (0.66–0.86)	< 0.001

a Model 1 adjusted for age and sex

b Model 2 adjusted for age, sex, coronary artery disease, atrial fibrillation, hypertension，diabetes mellitus, current smoking, current use of alcohol, year of enrollment, uric acid, serum creatinine, blood urea nitrogen, fasting glucose, triglycerides, low-density lipoprotein cholesterol, high density lipoprotein cholesterol, and Lipoprotein-associated phospholipase A2.

### 3.4 Association between Lp(a) levels and ALI by age and sex

In subgroup analyses stratified by age and sex, serum Lp(a) levels were consistently inversely correlated with ALI in all subgroups (all *P* < 0.05, Table 4). The magnitude of this inverse association was more pronounced among participants aged ≥ 60 years (Model 2: OR, 0.71; 95% CI, 0.60–0.84; *P* < 0.001) and among females (Model 2: 0.68; 95% CI, 0.53–0.88; *P* = 0.003) compared with those aged < 60 years (Model 2: OR, 0.76; 95% CI, 0.59–0.98; *P* = 0.034) and males (Model 2: OR, 0.80; 95% CI, 0.69–0.94; *P* = 0.007).

**Table 4:** **The association between Lp(a) levels with ALI by different age and sex**.

Variable	LgLp(a) ALI vs. control	Unadjusted OR (95% CI)	*P* value	Model 1^a^		Model 2^b^	
				Adjusted OR (95% CI)	*P* value	Adjusted OR (95% CI)	*P* value
Age							
< 60	2.04 ± 0.39 vs 2.12 ± 0.35	0.82 (0.70–0.96)	0.020	0.82 (0.70–0.96)	0.010	0.76 (0.59–0.98)	0.034
≥ 60	2.09 ± 0.39 vs 2.23 ± 0.35	0.65 (0.58–0.73)	< 0.001	0.66 (0.59–0.74)	< 0.001	0.71 (0.60–0.84)	< 0.001
Sex							
Male	2.05 ± 0.37 vs 2.16 ± 0.36	0.74 (0.67–0.83)	< 0.001	0.75 (0.67–0.83)	< 0.001	0.80 (0.69–0.94)	0.007
Female	2.11 ± 0.36 vs 2.27 ± 0.34	0.62 (0.51–0.74)	< 0.001	0.62 (0.51–0.74)	< 0.001	0.68 (0.53–0.88)	0.003

a Model 1 adjusted for age and sex

b Model 2 adjusted for age, sex, coronary artery disease, atrial fibrillation, hypertension，diabetes mellitus, current smoking, current use of alcohol, year of enrollment, uric acid, serum creatinine, blood urea nitrogen, fasting glucose, triglycerides, low-density lipoprotein cholesterol, high density lipoprotein cholesterol, and Lipoprotein-associated phospholipase A2.

## 4 Discussion

This study suggested that higher serum Lp(a) levels were independently and negatively correlated with the presence of ALI. Further analyses by infarct location revealed heterogeneity in this association across different brain regions: the strongest negative association was observed in the basal ganglia, while a weaker but still significant negative association was found in the cerebellum and brainstem. By contrast, the association in subcortical white matter infarcts did not reach statistical significance. Subgroup analyses confirmed that the inverse association was consistently observed across age and sex subgroups, with a more pronounced effect noted in participants aged 60 years or older and in women.

Our findings align with prior evidence suggesting a protective role of elevated serum Lp(a) against CSVD. In a Mendelian randomization study of a European cohort, Yuesong Pan et al. found that genetic variants of the 9SNP and 5SNP gene polymorphisms leading to higher Lp(a) concentrations were associated with a decrease in SAO(Pan et al., 2019), the most common underlying pathology of lacunar infarction. Our case-control results in a Chinese population are consistent with that observation, supporting the hypothesis that elevated Lp(a) may confer protection against CSVD-related ALI across different ethnic groups.

In contrast, some previous studies have reported either a null or a risk association between serum Lp(a) levels and ALI. Large-scale meta-analyses identified Lp(a) as a risk factor for all-cause ischemic stroke(Nave et al., 2015; Smolders et al., 2007), however, when stroke subtypes were examined separately, elevated Lp(a) posed risk for large artery atherosclerotic infarction, whereas no meaningful association was detected between Lp(a) and lacunar infarction or cerebral embolism(Kumar et al., 2021). A case-control study by Li Sun et al. even observed that each unit increase in Lp(a) was related to a two-fold increase in the odds of ALI, implying a deleterious effect of Lp(a) for ALI(Sun et al., 2003). The discrepancy may partly reflect differences in study design, population characteristics, and the distribution of Lp(a) levels.

The regional heterogeneity of the Lp(a)-ALI association is intriguing. The association we observed was strongest in the basal ganglia, weaker but still significant in the cerebellum/brainstem, and absent in the subcortical white matter. This gradient might reflect the regional differences in the predominant mechanisms underlying ALI. According to previous studies, the pathogenesis of ALI is multifactorial, with CSVD as the most important etiology, and other possible causes like branch atheromatous disease, parent artery atherosclerosis, arterial-to-arterial embolism, cardiogenic embolism, etc.(Regenhardt et al., 2018). The perforating arteries that supply the basal ganglia are typical cerebral small vessels and are highly vulnerable to CSVD; therefore, a protective effect of Lp(a) on small vessel pathology would be most evident in this region. In contrast, subcortical white matter arterioles represent the anatomical continuum of large cerebral arteries. Consequently, they are more susceptible to local arterial atherosclerosis, arterial-to-arterial embolism from unstable parent-artery plaques, and cardiogenic embolism – all of which are pathological processes generally promoted by elevated Lp(a) levels. Such detrimental mechanisms may thus partially counteract the potential protective effect of Lp(a) on CSVD-related lacunar infarction within the subcortical white matter, thereby accounting for the non-significant association observed in this region. The cerebral small vessels in the cerebellar and brainstem regions exhibit morphological features in-between those of the basal ganglia and subcortical white matter, and are a mixture of perforating branches and terminal branches. This anatomical characteristic explains the intermediate strength of the negative correlation between Lp(a) and ALI in the cerebellar and brainstem.

The mechanisms bridging Lp(a) and CSVD protection, though primarily derived from in vitro models, centers on its robust endothelial-stabilizing, anti-inflammatory, and anti-thrombotic properties. Lp(a) functions as a preferred carrier for oxidized phospholipids (OxPL)(Bergmark et al., 2008) which derives from oxidized LDL(Tselepis, 2018), and can facilitate the physiological detoxification of OxPL via lipoprotein-associated phospholipase A₂, thereby mitigating endothelial dysfunction (Xu et al., 2021, Yan et al., 2017). Moreover, Lp(a) shares structural similarities with fibronectin and its increase may facilitate endothelial repair by binding to multiple vascular matrix proteins at sites of intimal injury(Borth et al., 1991). Although its Apo(a) structure is similar to plasminogen(Loscalzo et al., 1990), it lacks enzymatic activity(Gabel and Koschinsky, 1995) and is presumed to inhibit fibrinolysis and promote thrombosis, nevertheless, existing studies indicated that such prothrombotic effects only emerge at extremely high t-PA concentrations (≥ 500 ng/mL)(Rijken et al., 2021), while t-PA is largely confined to nutrient micro-vessels rather than the large vessels(Levin and del Zoppo, 1994). On the contrary, the anti-thrombotic properties over the potential pro-thrombotic actions of Lp(a) have been validated. Blencowe et al. showed that Lp(a) exhibits significantly higher binding ability for platelet activating factor acetyl-hydrolase than LDL(Blencowe et al., 1995), enabling it to decompose pro-inflammatory and pro-thrombotic lipid mediators(Smiley et al., 1991; Stremler et al., 1991), thus generating anti-platelet, anti-inflammation, and anti-oxidation effects(Lordan et al., 2019; Tjoelker et al., 1995). More importantly, at physiological concentrations, evidence showed Lp(a) can inhibit platelet activation in a dose-dependent manner(Tsironis et al., 2004), particularly suppressing collagen-induced platelet aggregation, reducing the release of serotonin and thromboxane A2(Gries et al., 1996) via blocking the bind of fibrinogen to the αIIbβ3 integrin(Barre, 2004) and antagonizing the IIb subunit of the fibrinogen (GPIIb/IIIa) receptor (Barre, 2007). However, it remains uncertain whether these experimental mechanisms can be extrapolated to the in vivo setting at the physiological Lp(a) concentrations observed in our population.

Several limitations of this study should be considered. First, the case-control study precludes causal inference and is susceptible to unmeasured confounders (e.g., serum homocysteine and apolipoprotein B) and reverse causation. Second, the control group was recruited from a hospital-based population; compared with community-based controls, these participants were older and had higher prevalence of hypertension, diabetes, and hyperlipidemia, which narrowed between-group differences of atherosclerotic risk factors. While this attenuated the interference of certain confounders and improved the specificity of the association analysis between Lp(a) and ALI, it limits generalizability and may introduce selection bias. Finally, we collected patient data over more than five years using different reagent batches for testing Lp(a), which may have introduced inter-assay variability and potential measurement heterogeneity. However, adjusting for the year of enrollment as a categorical covariate did not materially alter the Lp(a)–ALI associations, indicating the robustness of our findings to such heterogeneity.

## 5 Conclusions

Our retrospective case-control research has revealed that serum Lp(a) shows an independent negative correlation with the presence of ALI. The clinical relevance and underlying mechanisms of the role of Lp(a) level on ALI requires further investigation.

## CRediT authorship contribution statement

**Xiangliang Chen:** Writing – review & editing, Writing – original draft, Investigation, Formal analysis, Data curation, Conceptualization. **Nihong Chen:** Writing – original draft, Validation, Formal analysis, Data curation, Conceptualization. **Lin Zhu:** Writing – review & editing, Methodology, Investigation. **Qing Huang:** Writing – review & editing, Validation, Formal analysis. **Fuping Jiang:** Writing – review & editing, Writing – original draft, Supervision, Resources, Project administration, Methodology, Funding acquisition, Conceptualization.

## Ethics declaration

The authors have NOT obtained informed consent from participants or their legal representatives. This is a retrospective case-control study based on existing medical record data only. No direct contact with individual patients was performed. The study protocol, including the waiver of individual informed consent, was reviewed and approved by the Ethics Committee of Nanjing First Hospital of Nanjing Medical University. Patient‑identifiable information was removed prior to data analysis to protect participant privacy. All procedures were performed in accordance with relevant ethical guidelines.

This study was performed in compliance with relevant laws, regulatory frameworks and guidelines where the research took place. This study was approved by the Ethics Committee of Nanjing First Hospital of Nanjing Medical University. (Approval No. KY20250611-KS-04)

## Funding

The present study is supported by the Cadre Healthcare Program of Jiangsu Provincial Health Commission (BJ23036).

## Declaration of Competing Interest

All authors declare no potential conflicts of interest with respect to the research, authorship, and publication of this article. The study was approved by the Ethics Committee of Nanjing First Hospital of Nanjing Medical University (Ethics approval number: KY20250611-KS-04) and conducted according to the principles of the Declaration of Helsinki.

## References

[bib1] Arauz A., Murillo L., Cantu C., Barinagarrementeria F., Higuera J. (2003). Prospective study of single and multiple lacunar infarcts using magnetic resonance imaging: risk factors, recurrence, and outcome in 175 consecutive cases. Stroke.

[bib2] Barre D.E. (2004). Apoprotein (A) antagonises THE GPIIB/IIIA receptor on collagen and adp-stimulated human platelets. Front. Biosci..

[bib3] Barre D.E. (2007). Arginyl-glycyl-aspartyl (RGD) epitope of human apolipoprotein (a) inhibits platelet aggregation by antagonizing the IIb subunit of the fibrinogen (GPIIb/IIIa) receptor. Thromb. Res..

[bib4] Bergmark C., Dewan A., Orsoni A., Merki E., Miller E.R., Shin M.J., Binder C.J., Horkko S., Krauss R.M., Chapman M.J., Witztum J.L., Tsimikas S. (2008). A novel function of lipoprotein [a] as a preferential carrier of oxidized phospholipids in human plasma. J. Lipid Res..

[bib5] Bhatia H.S., Wandel S., Willeit P., Lesogor A., Bailey K., Ridker P.M., Nestel P., Simes J., Tonkin A., Schwartz G.G., Colhoun H., Wanner C., Tsimikas S. (2025). Independence of Lipoprotein(a) and Low-Density Lipoprotein Cholesterol-Mediated Cardiovascular Risk: A Participant-Level Meta-Analysis. Circulation.

[bib6] Blencowe C., Hermetter A., Kostner G.M., Deigner H.P. (1995). Enhanced association of platelet-activating factor acetylhydrolase with lipoprotein (a) in comparison with low density lipoprotein. J. Biol. Chem..

[bib7] Borth W., Chang V., Bishop P., Harpel P.C. (1991). Lipoprotein (a) is a substrate for factor XIIIa and tissue transglutaminase. J. Biol. Chem..

[bib8] Byrne D.J., Jagroop I.A., Montgomery H.E., Thomas M., Mikhailidis D.P., Milton N.G., Winder A.F. (2002). Lipoprotein (a) does not participate in the early acute phase response to training or extreme physical activity and is unlikely to enhance any associated immediate cardiovascular risk. J. Clin. Pathol..

[bib9] Caplan L.R. (2015). Lacunar infarction and small vessel disease: pathology and pathophysiology. J. Stroke.

[bib10] Cerrato P., Imperiale D., Fornengo P., Bruno G., Cassader M., Maffeis P., Cavallo Perin P., Pagano G., Bergamasco B. (2002). Higher lipoprotein (a) levels in atherothrombotic than lacunar ischemic cerebrovascular disease. Neurology.

[bib11] Chen X., Wang L., Jiang J., Gao Y., Zhang R., Zhao X., Shen T., Dai Q., Li J. (2021). Association of neuroimaging markers of cerebral small vessel disease with short-term outcomes in patients with minor cerebrovascular events. BMC Neurol..

[bib12] Clarke R., Peden J.F., Hopewell J.C., Kyriakou T., Goel A., Heath S.C., Parish S., Barlera S., Franzosi M.G., Rust S., Bennett D., Silveira A., Malarstig A., Green F.R., Lathrop M., Gigante B., Leander K., de Faire U., Seedorf U., Hamsten A., Collins R., Watkins H., Farrall M., Consortium P. (2009). Genetic variants associated with Lp(a) lipoprotein level and coronary disease. N. Engl. J. Med..

[bib13] Duan Y., Zhao D., Sun J., Liu J., Wang M., Hao Y., Li J., Liu T., Xiao L., Hao Y., Wang H., Qi Y., Liu J. (2023). Lipoprotein(a) Is Associated With the Progression and Vulnerability of New-Onset Carotid Atherosclerotic Plaque. Stroke.

[bib14] European Heart Rhythm A., European Association for Cardio-Thoracic S., Camm A.J., Kirchhof P., Lip G.Y., Schotten U., Savelieva I., Ernst S., Van Gelder I.C., Al-Attar N., Hindricks G., Prendergast B., Heidbuchel H., Alfieri O., Angelini A., Atar D., Colonna P., De Caterina R., De Sutter J., Goette A., Gorenek B., Heldal M., Hohloser S.H., Kolh P., Le Heuzey J.Y., Ponikowski P., Rutten F.H. (2010). Guidelines for the management of atrial fibrillation: the Task Force for the Management of Atrial Fibrillation of the European Society of Cardiology (ESC). Eur. Heart J..

[bib15] Feng C., Bai X., Xu Y., Hua T., Huang J., Liu X.Y. (2013). Hyperhomocysteinemia associates with small vessel disease more closely than large vessel disease. Int. J. Med. Sci..

[bib16] Gabel B.R., Koschinsky M.I. (1995). Analysis of the proteolytic activity of a recombinant form of apolipoprotein(a). Biochemistry.

[bib17] Giroud M., Boutron M.C., Gras P., Gambert P., Lallemant C., Milan C., Essayagh E., Dumas R. (1992). Plasma lipoproteins in cortical versus lacunar infarction with or without cardiac arrhythmia, and in transient ischaemic attacks: a case control study. Neurol. Res..

[bib18] Glader C.A., Stegmayr B., Boman J., Stenlund H., Weinehall L., Hallmans G., Dahlen G.H. (1999). Chlamydia pneumoniae antibodies and high lipoprotein(a) levels do not predict ischemic cerebral infarctions. Results from a nested case-control study in Northern Sweden. Stroke.

[bib19] Gries A., Gries M., Wurm H., Kenner T., Ijsseldijk M., Sixma J.J., Kostner G.M. (1996). Lipoprotein(a) inhibits collagen-induced aggregation of thrombocytes. Arterioscler. Thromb. Vasc. Biol..

[bib20] Hobbs H.H., White A.L. (1999). Lipoprotein(a): intrigues and insights. Curr. Opin. Lipidol..

[bib21] Jackson C., Sudlow C. (2005). Are lacunar strokes really different? A systematic review of differences in risk factor profiles between lacunar and nonlacunar infarcts. Stroke.

[bib22] Jackson C.A., Hutchison A., Dennis M.S., Wardlaw J.M., Lindgren A., Norrving B., Anderson C.S., Hankey G.J., Jamrozik K., Appelros P., Sudlow C.L. (2010). Differing risk factor profiles of ischemic stroke subtypes: evidence for a distinct lacunar arteriopathy?. Stroke.

[bib23] Kargman D.E., Tuck C., Berglund L., Lin I.F., Mukherjee R.S., Thompson E.V., Jones J., Boden-Albala B., Paik M.C., Sacco R.L. (1998). Lipid and lipoprotein levels remain stable in acute ischemic stroke: the Northern Manhattan Stroke Study. Atherosclerosis.

[bib24] Koschinsky M.L., Cote G.P., Gabel B., van der Hoek Y.Y. (1993). Identification of the cysteine residue in apolipoprotein(a) that mediates extracellular coupling with apolipoprotein B-100. J. Biol. Chem..

[bib25] Kronenberg F. (2014). Lipoprotein(a) in various conditions: to keep a sense of proportions. Atherosclerosis.

[bib26] Kronenberg F., Mora S., Stroes E.S.G., Ference B.A., Arsenault B.J., Berglund L., Dweck M.R., Koschinsky M., Lambert G., Mach F., McNeal C.J., Moriarty P.M., Natarajan P., Nordestgaard B.G., Parhofer K.G., Virani S.S., von Eckardstein A., Watts G.F., Stock J.K., Ray K.K., Tokgozoglu L.S., Catapano A.L. (2022). Lipoprotein(a) in atherosclerotic cardiovascular disease and aortic stenosis: a European Atherosclerosis Society consensus statement. Eur. Heart J..

[bib27] Kumar P., Swarnkar P., Misra S., Nath M. (2021). Lipoprotein (a) level as a risk factor for stroke and its subtype: A systematic review and meta-analysis. Sci. Rep..

[bib28] Langsted A., Nordestgaard B.G., Kamstrup P.R. (2019). Elevated Lipoprotein(a) and Risk of Ischemic Stroke. J. Am. Coll. Cardiol..

[bib29] Larsson S.C., Gill D., Mason A.M., Jiang T., Back M., Butterworth A.S., Burgess S. (2020). Lipoprotein(a) in Alzheimer, atherosclerotic, cerebrovascular, thrombotic, and valvular disease: mendelian randomization investigation. Circulation.

[bib30] Levin E.G., del Zoppo G.J. (1994). Localization of tissue plasminogen activator in the endothelium of a limited number of vessels. Am. J. Pathol..

[bib31] Lordan R., Tsoupras A., Zabetakis I., Demopoulos C.A. (2019). Forty years since the structural elucidation of platelet-activating factor (PAF): historical, current, and future research perspectives. Molecules.

[bib32] Loscalzo J., Weinfeld M., Fless G.M., Scanu A.M. (1990). Lipoprotein(a), fibrin binding, and plasminogen activation. Arteriosclerosis.

[bib33] Markus H.S., de Leeuw F.E. (2023). Cerebral small vessel disease: recent advances and future directions. Int. J. Stroke.

[bib34] Milionis H.J., Winder A.F., Mikhailidis D.P. (2000). Lipoprotein (a) and stroke. J. Clin. Pathol..

[bib35] Nave A.H., Lange K.S., Leonards C.O., Siegerink B., Doehner W., Landmesser U., Steinhagen-Thiessen E., Endres M., Ebinger M. (2015). Lipoprotein (a) as a risk factor for ischemic stroke: a meta-analysis. Atherosclerosis.

[bib36] Pan Y., Li H., Wang Y., Meng X., Wang Y. (2019). Causal Effect of Lp(a) [Lipoprotein(a)] Level on ischemic stroke and Alzheimer disease: a mendelian randomization study. Stroke.

[bib37] Petersen N.H., Schmied A.B., Zeller J.A., Plendl H., Deuschl G., Zunker P. (2007). Lp(a) lipoprotein and plasminogen activity in patients with different etiology of ischemic stroke. Cerebrovasc. Dis..

[bib38] Price J.F., Lee A.J., Rumley A., Lowe G.D., Fowkes F.G. (2001). Lipoprotein (a) and development of intermittent claudication and major cardiovascular events in men and women: the Edinburgh Artery Study. Atherosclerosis.

[bib39] Regenhardt R.W., Das A.S., Lo E.H., Caplan L.R. (2018). Advances in Understanding the Pathophysiology of Lacunar Stroke: A Review. JAMA Neurol..

[bib40] Ridker P.M., Stampfer M.J., Hennekens C.H. (1995). Plasma concentration of lipoprotein(a) and the risk of future stroke. JAMA.

[bib41] Rijken D.C., de Vries J.J., Malfliet J., Bos S., Kronenberg F., Leijten F.P., Roeters van Lennep J.E., Uitte de Willige S., van der Zee L., Mulder M.T. (2021). How significant is the antifibrinolytic effect of lipoprotein(a) for blood clot lysis?. Thromb. Res..

[bib42] Schilling S., Tzourio C., Dufouil C., Zhu Y., Berr C., Alperovitch A., Crivello F., Mazoyer B., Debette S. (2014). Plasma lipids and cerebral small vessel disease. Neurology.

[bib43] Shi Y., Guo L., Chen Y., Xie Q., Yan Z., Liu Y., Kang J., Li S. (2021). Risk factors for ischemic stroke: differences between cerebral small vessel and large artery atherosclerosis aetiologies. Folia Neuropathol..

[bib44] Shu M.J., Zhai F.F., Zhang D.D., Han F., Zhou L., Ni J., Yao M., Zhang S.Y., Cui L.Y., Jin Z.Y., Zhu H.J., Zhu Y.C. (2021). Metabolic syndrome, intracranial arterial stenosis and cerebral small vessel disease in community-dwelling populations. Stroke Vasc. Neurol..

[bib45] Smiley P.L., Stremler K.E., Prescott S.M., Zimmerman G.A., McIntyre T.M. (1991). Oxidatively fragmented phosphatidylcholines activate human neutrophils through the receptor for platelet-activating factor. J. Biol. Chem..

[bib46] Smolders B., Lemmens R., Thijs V. (2007). Lipoprotein (a) and stroke: a meta-analysis of observational studies. Stroke.

[bib47] Stremler K.E., Stafforini D.M., Prescott S.M., McIntyre T.M. (1991). Human plasma platelet-activating factor acetylhydrolase. Oxidatively fragmented phospholipids as substrates. J. Biol. Chem..

[bib48] Sun L., Li Z., Zhang H., Ma A., Liao Y., Wang D., Zhao B., Zhu Z., Zhao J., Zhang Z., Wang W., Hui R. (2003). Pentanucleotide TTTTA repeat polymorphism of apolipoprotein(a) gene and plasma lipoprotein(a) are associated with ischemic and hemorrhagic stroke in Chinese: a multicenter case-control study in China. Stroke.

[bib49] Tang Y., Geng D. (2019). Associations of plasma LP(a), Hcy and D-D levels with the subtype of ischemic cerebrovascular disease. Med. (Baltim. ).

[bib50] Tjoelker L.W., Wilder C., Eberhardt C., Stafforini D.M., Dietsch G., Schimpf B., Hooper S., Le Trong H., Cousens L.S., Zimmerman G.A., Yamada Y., McIntyre T.M., Prescott S.M., Gray P.W. (1995). Anti-inflammatory properties of a platelet-activating factor acetylhydrolase. Nature.

[bib51] Tselepis A.D. (2018). Oxidized phospholipids and lipoprotein-associated phospholipase A(2) as important determinants of Lp(a) functionality and pathophysiological role. J. Biomed. Res..

[bib52] Tsironis L.D., Mitsios J.V., Milionis H.J., Elisaf M., Tselepis A.D. (2004). Effect of lipoprotein (a) on platelet activation induced by platelet-activating factor: role of apolipoprotein (a) and endogenous PAF-acetylhydrolase. Cardiovasc. Res..

[bib53] van Dam-Nolen D.H.K., van Dijk A.C., Crombag G., Lucci C., Kooi M.E., Hendrikse J., Nederkoorn P.J., Daemen M., van der Steen A.F.W., Koudstaal P.J., Kronenberg F., Roeters van Lennep J.E., Mulder M.T., van der Lugt A. (2021). Lipoprotein(a) levels and atherosclerotic plaque characteristics in the carotid artery: The Plaque at RISK (PARISK) study. Atherosclerosis.

[bib54] Wang L., Gao P., Zhang M., Huang Z., Zhang D., Deng Q., Li Y., Zhao Z., Qin X., Jin D., Zhou M., Tang X., Hu Y., Wang L. (2017). Prevalence and ethnic pattern of diabetes and prediabetes in China in 2013. JAMA.

[bib55] Wang Z., Chen Z., Zhang L., Wang X., Hao G., Zhang Z., Shao L., Tian Y., Dong Y., Zheng C., Wang J., Zhu M., Weintraub W.S., Gao R., China Hypertension Survey I. (2018). Status of Hypertension in China: results from the China Hypertension Survey, 2012-2015. Circulation.

[bib56] Wardlaw J.M. (2005). What causes lacunar stroke?. J. Neurol. Neurosurg. Psychiatry.

[bib57] Xu L., Schooling C.M., Chan W.M., Lee S.Y., Leung G.M., Lam T.H. (2013). Smoking and hemorrhagic stroke mortality in a prospective cohort study of older Chinese. Stroke.

[bib58] Xu S., Ilyas I., Little P.J., Li H., Kamato D., Zheng X., Luo S., Li Z., Liu P., Han J., Harding I.C., Ebong E.E., Cameron S.J., Stewart A.G., Weng J. (2021). Endothelial dysfunction in atherosclerotic cardiovascular diseases and beyond: from mechanism to pharmacotherapies. Pharmacol. Rev..

[bib59] Yan F.X., Li H.M., Li S.X., He S.H., Dai W.P., Li Y., Wang T.T., Shi M.M., Yuan H.X., Xu Z., Zhou J.G., Ning D.S., Mo Z.W., Ou Z.J., Ou J.S. (2017). The oxidized phospholipid POVPC impairs endothelial function and vasodilation via uncoupling endothelial nitric oxide synthase. J. Mol. Cell. Cardiol..

[bib60] Zeng M.D., Li Y.M., Chen C.W., Lu L.G., Fan J.G., Wang B.Y., Mao Y.M., Chinese National Consensus Workshop on Nonalcoholic Fatty Liver D. (2008). Guidelines for the diagnosis and treatment of alcoholic liver disease. J. Dig. Dis..

